# Neurofilament light and heterogeneity of disease progression in amyotrophic lateral sclerosis: development and validation of a prediction model to improve interventional trials

**DOI:** 10.1186/s40035-021-00257-y

**Published:** 2021-08-26

**Authors:** Simon Witzel, Felix Frauhammer, Petra Steinacker, David Devos, Pierre-François Pradat, Vincent Meininger, Steffen Halbgebauer, Patrick Oeckl, Joachim Schuster, Simon Anders, Johannes Dorst, Markus Otto, Albert C. Ludolph

**Affiliations:** 1 Department of Neurology, University of Ulm, Ulm, Germany; 2 Center for Molecular Biology, Heidelberg University, Heidelberg, Germany; 3 Department of Medical Pharmacology, Expert center for Parkinson, CHU-Lille, Lille Neuroscience and Cognition, Inserm, UMR-S1172, LICEND, NS-Park Network, University of Lille, Lille, France; 4 Laboratoire D’Imagerie Biomédicale, Sorbonne Université, CNRS, INSERM, Paris, France; 5 APHP, Département de Neurologie, Hôpital Pitié-Salpêtrière, Paris, France; 6 German Centre for Neurodegenerative Diseases (DZNE) Site Ulm, Ulm, Germany

**Keywords:** Neurofilament light, Prediction model, Disease progression, Amyotrophic lateral sclerosis, Interventional trials, Statistical power

## Abstract

**Background:**

Interventional trials in amyotrophic lateral sclerosis (ALS) suffer from the heterogeneity of the disease as it considerably reduces statistical power. We asked if blood neurofilament light chains (NfL) could be used to anticipate disease progression and increase trial power.

**Methods:**

In 125 patients with ALS from three independent prospective studies—one observational study and two interventional trials—we developed and externally validated a multivariate linear model for predicting disease progression, measured by the monthly decrease of the ALS Functional Rating Scale Revised (ALSFRS-R) score. We trained the prediction model in the observational study and tested the predictive value of the following parameters assessed at diagnosis: NfL levels, sex, age, site of onset, body mass index, disease duration, ALSFRS-R score, and monthly ALSFRS-R score decrease since disease onset. We then applied the resulting model in the other two study cohorts to assess the actual utility for interventional trials. We analyzed the impact on trial power in mixed-effects models and compared the performance of the NfL model with two currently used predictive approaches, which anticipate disease progression using the ALSFRS-R decrease during a three-month observational period (lead-in) or since disease onset (ΔFRS).

**Results:**

Among the parameters provided, the NfL levels (*P* < 0.001) and the interaction with site of onset (*P* < 0.01) contributed significantly to the prediction, forming a robust NfL prediction model (*R* = 0.67). Model application in the trial cohorts confirmed its applicability and revealed superiority over lead-in and ΔFRS-based approaches. The NfL model improved statistical power by 61% and 22% (95% confidence intervals: 54%–66%, 7%–29%).

**Conclusion:**

The use of the NfL-based prediction model to compensate for clinical heterogeneity in ALS could significantly increase the trial power.

NCT00868166, registered March 23, 2009; NCT02306590, registered December 2, 2014.

**Supplementary Information:**

The online version contains supplementary material available at 10.1186/s40035-021-00257-y.

## Introduction

The Amyotrophic Lateral Sclerosis Functional Rating Scale Revised (ALSFRS-R) score has become the predominantly used primary outcome parameter in ALS trials [1, 2]. The ALSFRS-R assesses the functional capability of ALS patients in daily life, and the score points lost per month are an established parameter for disease progression rate [3]. Interventional trials use the score to investigate if a treatment slows down the functional decline.

**Fig. 1:**
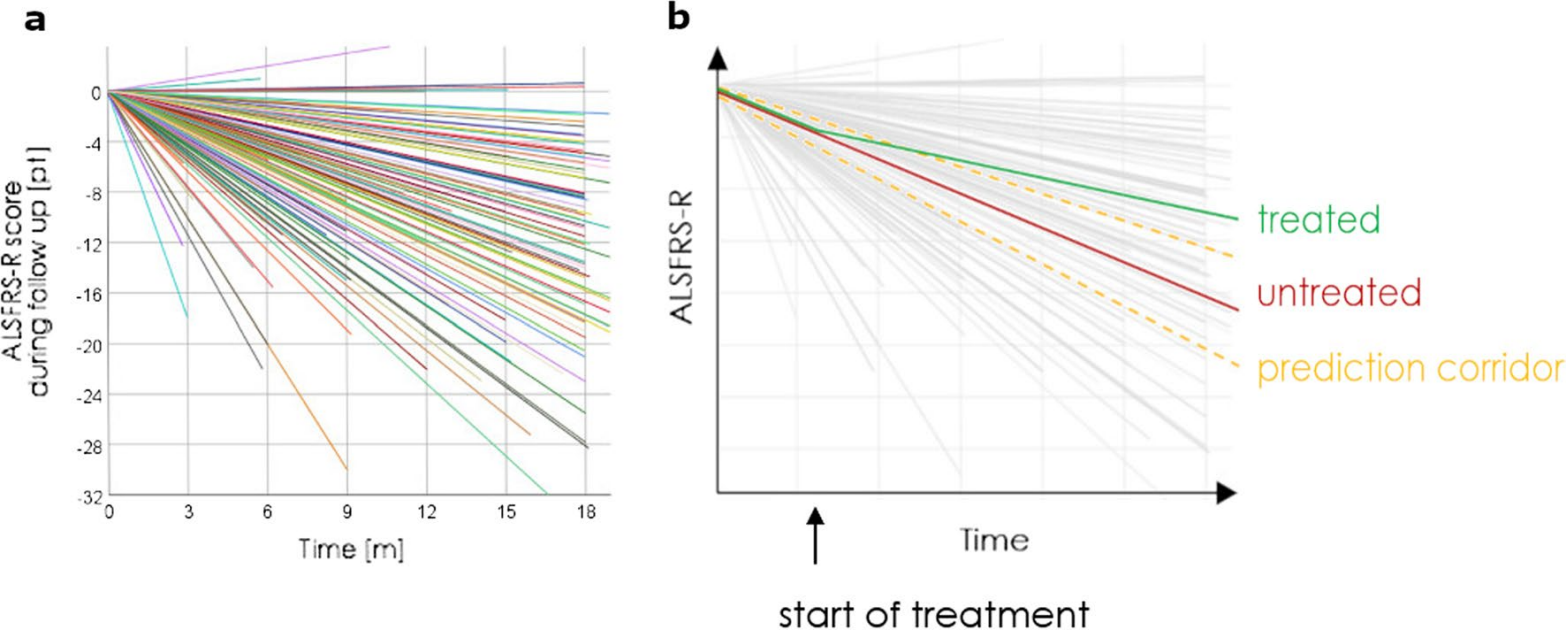
Heterogeneity of disease progression rates and possible application of a prediction model. **a** The heterogeneity of disease progression rates in ALS, as shown by ALSFRS-R slopes of each study participant in the three cohorts of this study during the follow-up time. **b** The application of a prediction model in one patient receiving an efficient treatment. Note that without the use of a prediction model, the treatment effect (difference between the red and green lines) can hardly be differentiated from natural heterogeneity. Eventually, the use of the prediction model (yellow lines) reveals a significant slowdown of disease progression

Due to the heterogeneity of the disease, the progression rates vary greatly between patients [4–7]. Figure 1a shows the variability of progression rates in an example of our trial participants. The interindividual differences hamper the ability to recognize a treatment effect and thereby reduce the statistical power. Therefore, the disease heterogeneity is a major challenge in the design of ALSFRS-R-based trials [8–10]. The power issues have been discussed in some positive ALSFRS-R-based phase 2 trials, of which the positive results could not be reproduced in phase 3, as well as in the context of many negative trials in ALS [8, 11–15]. Due to the low prevalence of ALS, the heterogeneity cannot easily be compensated by increasing the number of trial participants [16]. Using prediction models to anticipate the patients' disease progression rates throughout a trial is considered a promising strategy to meet this challenge [17–21] (see also Fig. 1b).

However, there is still a lack of sufficiently validated prediction models for the ALSFRS-R course, and thus no implementation of such models in randomized controlled trials to date. Instead of using prediction models, current clinical trials have measured the ALSFRS-R decrease during an observational phase of several months in the enrolment process or used the monthly decrease of ALSFRS-R score since disease onset (ΔFRS) to estimate a patient's disease progression.

Neurofilaments are increasingly recognized as a prognostic biomarker for ALS [22, 23]. For blood levels of neurofilament light chains (NfLs), a moderate correlation with disease progression rate has repeatedly been reported [24–28]. The good accessibility, objectivity, and prognostic value have made NfL a promising candidate biomarker to improve prediction models.

In this study, we set out to study if NfL levels could be used to improve prognostic models, and evaluate the transferability of the prediction models to new datasets to test their practical applications and to quantify the potential impact on trial power.

## Materials and methods

### Study cohorts

**Fig. 2:**
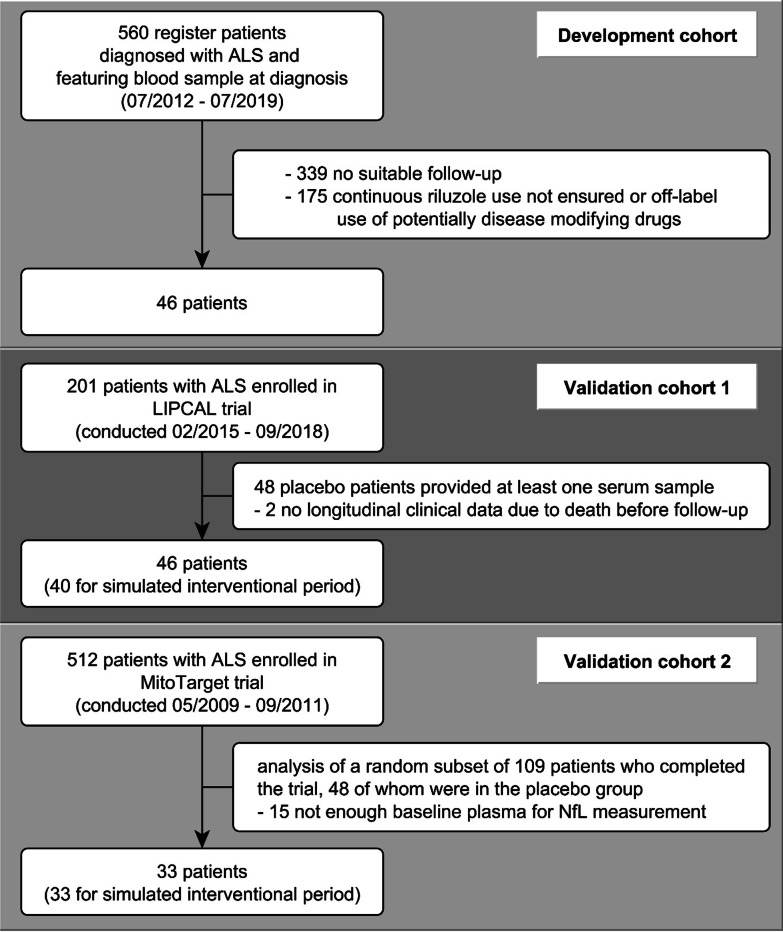
Flowchart of participant inclusion from three cohorts

This study included three independent ALS cohorts, an observational cohort for model development (DC) and two trial cohorts for model validation (V1 and V2) (Fig. 2).

The DC cohort consists of patients with ALS who participated in an observational study at the Department of Neurology at Ulm University (German Network for Motor Neuron Diseases, MND-NET site Ulm, Ulm, Germany). At the time of analysis, the observational study included prospective clinical parameters and biosamples of 1440 patients collected between 07/2012 and 07/2019, of whom 560 had an available blood sample at diagnosis. To ensure a trial-like longitudinal cohort and the broadest possible progression spectrum for model development, we defined the following eligibility criteria: patients at their time of diagnosis with a probable (clinically or laboratory-supported) or definite ALS according to the revised version of the El Escorial criteria [29], with follow-up blood sampling between 5 and 12 months after diagnosis. Also, the patients needed a documented continuous riluzole treatment since the use of riluzole is an inclusion criterion in most interventional trials. Treatments with edaravone, rasagiline, or high-caloric nutritional supplements were defined as exclusion criteria due to potential disease-modifying effects [30–32].

The validation cohorts were acquired from the placebo arms of two completed ALS trials. V1 consisted of patients on placebo who participated in LIPCAL ALS, a trial investigating a high caloric fatty diet (conducted between 02/2015 and 09/2018; *n* = 201; follow-up time: 18 months) [32, 33]. In this study, serum was collected on a voluntary basis, resulting in blood sample availability in 46 patients [33]. V2 consisted of patients who participated in the MitoTarget ALS trial investigating olesoxime, a drug interacting with mitochondrial membrane proteins and associated with neuroprotective features (conducted between 05/2009 and 09/2011; *n* = 512; follow-up time: 18 months) [19, 34]. We used EDTA plasma samples from a randomly selected subgroup of patients who had completed the 18-month follow-up, equivalent to 33 patients on placebo [19]. In both trials, the patients were continuously treated with riluzole.

### Model development

The prediction model was developed in the DC cohort in patients at their time of diagnosis using multiple linear regression, with the subsequent decrease of ALSFRS-R score per month (ALSFRS-R slope; pt/m) as the dependent variable. We investigated the predictive value of blood NfL levels at the time of diagnosis and the clinical parameters sex, age, site of onset (bulbar or spinal), body mass index, disease duration, monthly ALSFRS-R decrease since disease onset (ΔFRS), and ALSFRS-R score at diagnosis as independent variables. NfL was logarithmically transformed to ln(NfL) to achieve a normal distribution.

To determine the predictive quality of the candidate predictors, we compared all possible combinations of candidate predictors using a sequence of F-Tests. To identify the variables for the final prediction model, we eliminated in a one-by-one manner the non-significant variables with the largest *P*-value in the coefficient analysis until only variables remained that statistically significantly contributed to the prediction.

### Model validation

For external model validation in the two validation cohorts, we used the patients' baseline data and our model to predict each patient's future ALSFRS-R slope. After this, we evaluated the absolute deviation between the predicted ALSFRS-R slopes and the ALSFRS-R slopes observed during the trial follow-up time, and visually checked for accuracy and systematic deviations.

In the second step, we split the follow-up time in the validation cohorts and separately computed ALSFRS-R slopes for the first three months of the study period mimicking an observational period (lead-in) and the subsequent time mimicking an interventional period. The splitting allowed us to compare the ALSFRS-R slopes during the interventional period with ALSFRS-R slopes predicted for each patient in three different ways: (1) using our prediction model with NfL levels and clinical parameters assessed at study baseline, (2) using ΔFRS, and (3) using the ALSFRS-R slope during the lead-in period.

The predictive quality of each prediction method was evaluated using established statistical methods: root-mean-square error (RMSE), Coefficient of Determination (CoefD), and variance change (see Statistical methods).

Finally, we analyzed each method's impact on statistical power using an approach based on mixed-effects models introduced by Küffner et al*.* [18] (see Statistical methods). Briefly, it computes the hypothetical reduction in trial size that could be compensated by normalizing on the disease progression rates derived from a predictive model. Using this method, trial size reduction becomes a measure for the increase in statistical power.

### NfL assay

Blood samples were obtained from peripheral blood and stored with strict adherence to standard operating procedures [35]. NfL concentrations in serum (DC and V1) or EDTA-plasma (V2) were measured in the same laboratory (Department of Neurology, University of Ulm, Germany) on a SIMOA HD-1 analyzer, using a commercially available kit (Quanterix, Lexington, MA) with an analytical limit of detection of 0.038 pg/ml given by the manufacturer, equally usable for serum and EDTA-plasma. Temporal fluctuations of ln(NfL) levels were studied by relative deviations from the patient's mean ln(NfL) value.

### Statistical methods

All statistical analyses were done in R version 4.0.0 using R packages lme4 (version 1.1.23), tidyverse (version 1.3.0), and cowplot (version 1.0.0) at a level of significance of *P* < 0.05.

The ALSFRS-R slopes in the DC were computed by linear regression, using all ALSFRS-R scores of a patient and their corresponding times since disease onset. The trial ALSFRS-R slopes were computed using linear regression with a patient's ALSFRS-R scores assessed throughout the trial and trial duration. The ΔFRS was computed using the formula:

1 $$\Delta FRS=\frac{48 - ALSFRS-R\, Score\, at\, baseline}{months\, between\, disease\, onset\, and\, baseline}$$

with 48 being the maximum ALSFRS-R score.

Goodness-of-fit for the internal validation was measured as the adjusted *R*^2^ and its square root *R*. For external validation, RMSE, CoefD, and variance change were computed with the following formulas:

2 $$\text{RMSE}=\sqrt{\left(mean\left(y-p\right)\right)}$$

3 $$\text{CoefD}=1-\sum \left({\left(y-p\right)}^{2}\right)/\sum \left({\left(y-m\right)}^{2}\right)$$

4 $$\text{Variance change}=\frac{\left( var \left(y-p\right)-var \left(y\right) \right)}{var (y)}$$

where *p* is the predicted ALSFRS-R slope, *y* the ALSFRS-R slope in the interventional period, and *m* the mean of *y*. Trend lines and standard error in Fig. 5 were calculated using ggplot2's *geom_smooth* function with *method* = *`lm`*.

To compute the trial size savings for a randomized, placebo-controlled clinical trial, we adopted the approach by Küffner et al*.* [18]. We randomly assigned patients from the validation cohorts (V1/V2) to treatment and control groups with equal sizes, retained only timepoints from the interventional period, and centered time to 0 at the start of the interventional period for each patient. We then fitted the following multivariable mixed-effects model for one cohort at a time and using the predictions from the NfL model, the lead-in period and ΔFRS:

5 $${a}_{ij} = {{a}_{i1} + \beta }_{0} + {b}_{i}{t}_{ij} + {\beta }_{1}{t}_{ij} + {\beta }_{2}{t}_{ij}treatmen{t}_{i} + {\beta }_{3}{p}_{i} + {\beta }_{4}{p}_{i}{t}_{ij}$$

where *a*_*ij*_ is the ALS-FRS-R value of patient *i* at time point *j*, *a*_*i*1_ is the first time point used as model offset, *t*_*ij*_ is the time since baseline, *treatment*_*i*_ is 0 (control) or 1 (treatment), and *p*_*i*_ is the predicted progression rate. In this model, *β*_0_ is the global intercept, *β*_1_ is the slope over time with a random effect per patient *b*_*i*_, *β*_2_ is the coefficient measuring the treatment effect and *β*_3_ and *β*_4_ are coefficients for the predicted slope.

The standard error of *β*_2_ was used to compare models for their statistical power to detect treatment effects using the formula

6 $$100\times\left(1-\left(S{E}_{alt}/S{E}_{null}\right)^{2}\right)$$

where *SE*_*alt*_ is the standard error of *β*_2_ in the above model, and *SE*_*null*_ is the standard error in a reduced model lacking all terms involving predictive information *p*_*i*_. To account for each patient's random assignment to a placebo or treatment group in the mixed-effects models, we applied each model in 10,000 permutations per predictive method and cohort. The reported 95% CIs are Monte Carlo CIs, i.e. the 2.5% and 97.5% quantiles across these permutations.

## Results

### Patient characteristics

In the observational study, 46 patients were eligible for model development. ALS was diagnosed at median 10.1 months (interquartile range [IQR] 6.51–19.4) after symptom onset at an ALSFRS-R score of 42 points (IQR 39.8–44.0). During a median follow-up time of 13.1 months (IQR 6.8–19.5) we observed a median ALSFRS-R slope of -0.73 pt/month (IQR -0.34 to -1.16). The composition of the validation cohorts was predefined by the eligibility criteria of the corresponding studies and the availability of blood samples for NfL measurement (flow chart, see Fig. 2). Table 1 displays the patient characteristics of the three cohorts.

**Table 1:** Patient characteristics

Cohort	Development cohort (*n* = 46)	Validation 1 (*n* = 46)	Validation 2 (*n* = 33)
Male/Female sex, *n* (%)	27 (59)/19 (41)	32 (65)/14 (35)	23 (70)/10 (30)
Spinal/Bulbar onset, *n* (%)	36 (78)/10 (22)	35 (71)/14 (29)	26 (79)/7 (21)
Age at disease onset, mean (SD), years	60.1 (11.3)	61.9 (10.4)	49.5 (11.7)
Age at baseline, mean (SD), years	61.3 (11.6)	63.4 (10.2)	51.3 (11.7)
Disease duration at baseline, median (IQR), months	10.1 (6.51–19.4)	14.8 (9.4–28.4)	20 (13.0–29.5)
ALSFRS-R Score at baseline, median (IQR), points	42 (39.8–44.0)	37 (30.5–42.0)	39 (34.0–43.0)
ΔFRS, median (IQR), -pt/m	0.56 (0.26–0.98)	0.60 (0.34–1.15)	0.44 (0.28–0.76)
BMI at baseline, median (IQR), kg/m^2^	25.4 (24.1–28.8)	24.0 (22.6–26.7)	23.8 (22.4–26.6)
Follow-up time, median (IQR), months	13.1 (6.8–19.5)	13.0 (9.0–18.0)	18.0 (18.0–18.0)
ALSFRS-R slope in entire follow-up, median (IQR), -pt/m	0.73 (0.34–1.16)	1.02 (0.48–1.55)	0.61 (0.32–0.86)
ALSFRS-R slope in interventional period, median (IQR), -pt/m		0.83 (0.49–1.49); *n* = 40	0.50 (0.25–0.87); *n* = 33
Baseline NfL levels, median (IQR), pg/ml	115 (64–174)	94 (54–141)	54 (33–86)
Baseline ln(NfL) levels, mean (SD), pg/ml	4.63 (0.81)	4.58 (0.83)	3.95 (0.66)

The table shows the patient characteristics of the three cohorts used for model development and validation. No ALSFRS-R slope in interventional period is specified for the development cohort, as this is not an interventional trial cohort

### NfL and its interaction with site of disease onset enable robust predictions

**Fig. 3:**
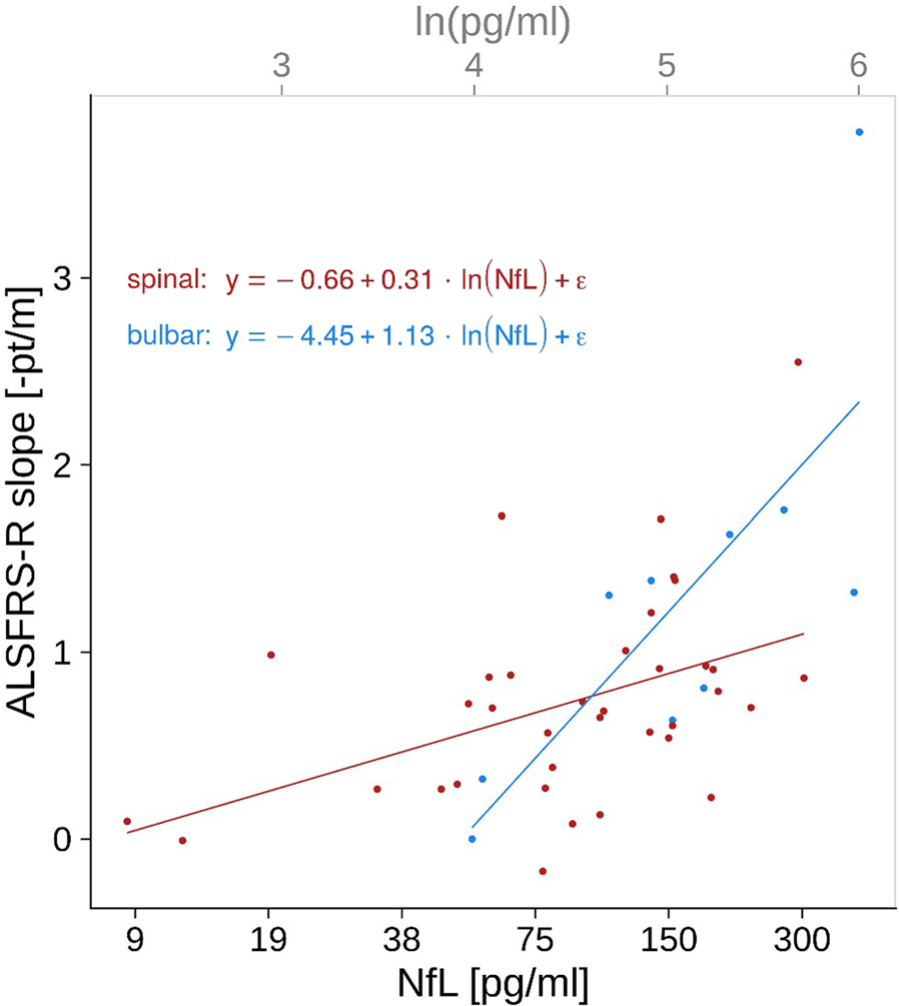
Scatter plot of disease progression rates (defined as ALSFRS-R slopes) and NfL blood levels at diagnosis. The NfL model formulas and corresponding regression lines derived from the multivariate regression in the development cohort are shown separately for patients with spinal (red) and bulbar onset (blue), to visualize the correlation between ALSFRS-R slopes and ln(NfL) levels and the interaction between ln(NfL) levels and site of onset

Multivariate regression, including all candidate predictors in the development cohort, showed that the ALSFRS-R slopes were significantly correlated with the ln(NfL) values (*P* < 0.001) and their interaction with site of onset (*P* < 0.01). Higher ln(NfL) levels were indicative of faster disease progression, and the interaction with site of onset resulted in a greater change of the ALSFRS-R slope per change of one NfL log unit in bulbar-onset patients compared to spinal-onset patients (Fig. 3). The candidate predictors sex, age, body mass index, disease duration, ΔFRS, and ALSFRS-R score at diagnosis did not add significantly to the prediction and hence were not included in the final model. By testing all possible compositions of predictors in multivariate linear regression models, we found that the models including ln(NfL) always outperformed corresponding models without ln(NfL).

The final NfL model had the following form, where *S* is the site of onset (*S* = 1 for spinal, *S* = 0 for bulbar):

7 $$\text{ALSFRS-R slope}=4.45 - 1.13 \ln (\text{NfL}) - 3.82\,S + 0.83\,S \ln (\text{NfL})$$

Applying this formula, an NfL value of 100 pg/ml results in an ALSFRS-R slope of − 0.75 pt/m — at this value, patients with a spinal disease onset and those with a bulbar disease onset would have an identical ALSFRS-R slope. The model predicts a ± 0.5 pt/m change of progression rate by an NfL level change of ± 1.67 logarithmic units for patients with spinal onset and ± 0.44 logarithmic units for patients with bulbar onset, respectively.

Internal validation of the model showed a correlation of *R* = 0.67 between predicted and measured ALSFRS-R slopes. We verified the model, applying the same development process in the validation cohorts; this led us to the same significant predictors showing similarly high correlations (*R* = 0.65 and 0.62). Including the site of onset significantly improved model performance compared to using ln(NfL) as the only predictor in each cohort.

### NfL levels are stable over time

**Fig. 4:**
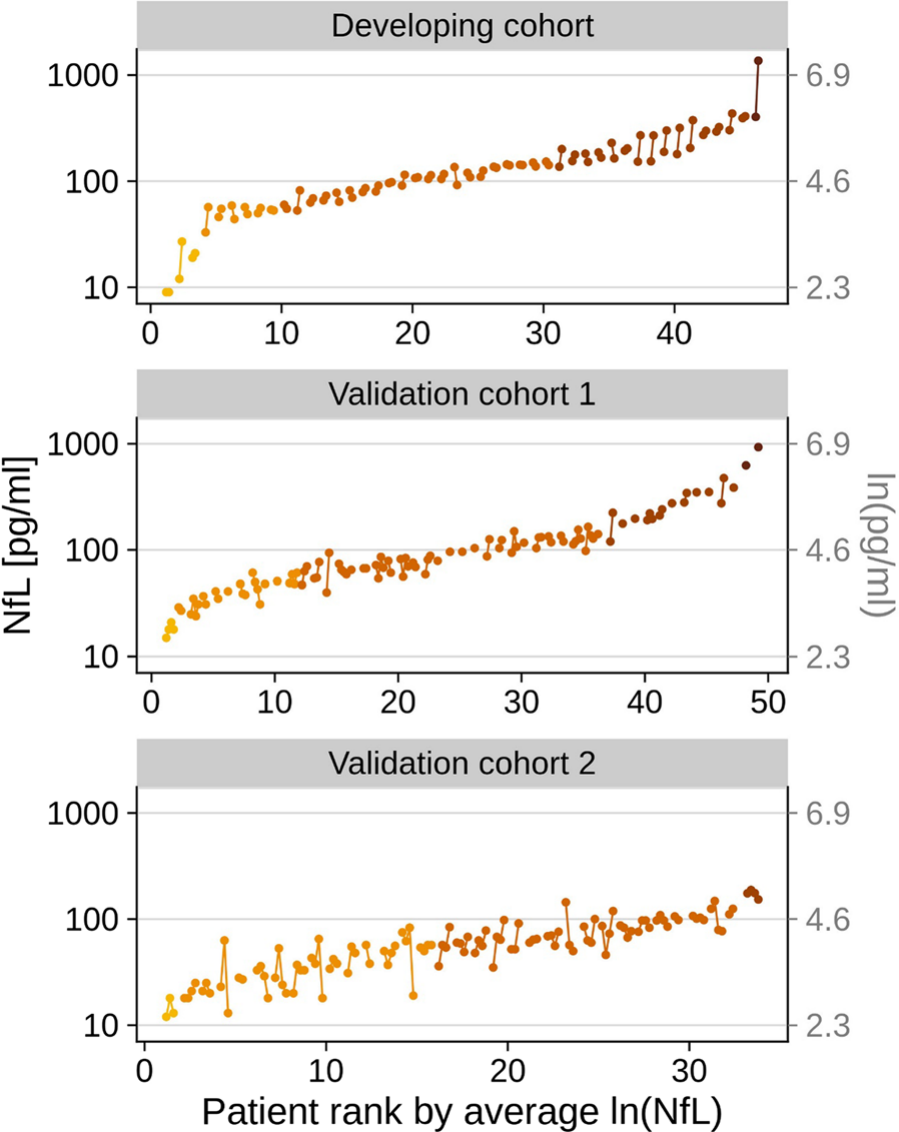
Temporal fluctuations of NfL blood levels in each patient. ln(NfL) measurements from each individual patient are connected by lines, and patients are colored based on the order of magnitude of their average ln(NfL) values. The time points of a given patient are ordered by time from left to right and equally spaced

To assess the temporal stability of ln(NfL) measurements for each patient, we visualized the trajectories for all three cohorts (Fig. 4). Measurements from different patients ranged over multiple orders of magnitude, while measurements from the same patient showed comparatively small variation. Importantly, these variations were small compared to the patient's average ln(NfL) value, with mean relative deviations (± SD) in the DC/V1/V2 cohorts of 2.5% (± 3.7%), 3.0% (± 3.8%), and 4.8% (± 7.2%). The 2.5 and 97.5 percentiles for the absolute difference between the baseline ln(NfL) levels and the ln(NfL) levels at the first follow-up for the DC/V1/V2 cohorts were: [− 0.33, 0.79], [− 0.37, 0.60], [− 0.93, 0.85] log units.

### The NfL prediction model is transferable to actual clinical trials and increases statistical power

**Fig. 5:**
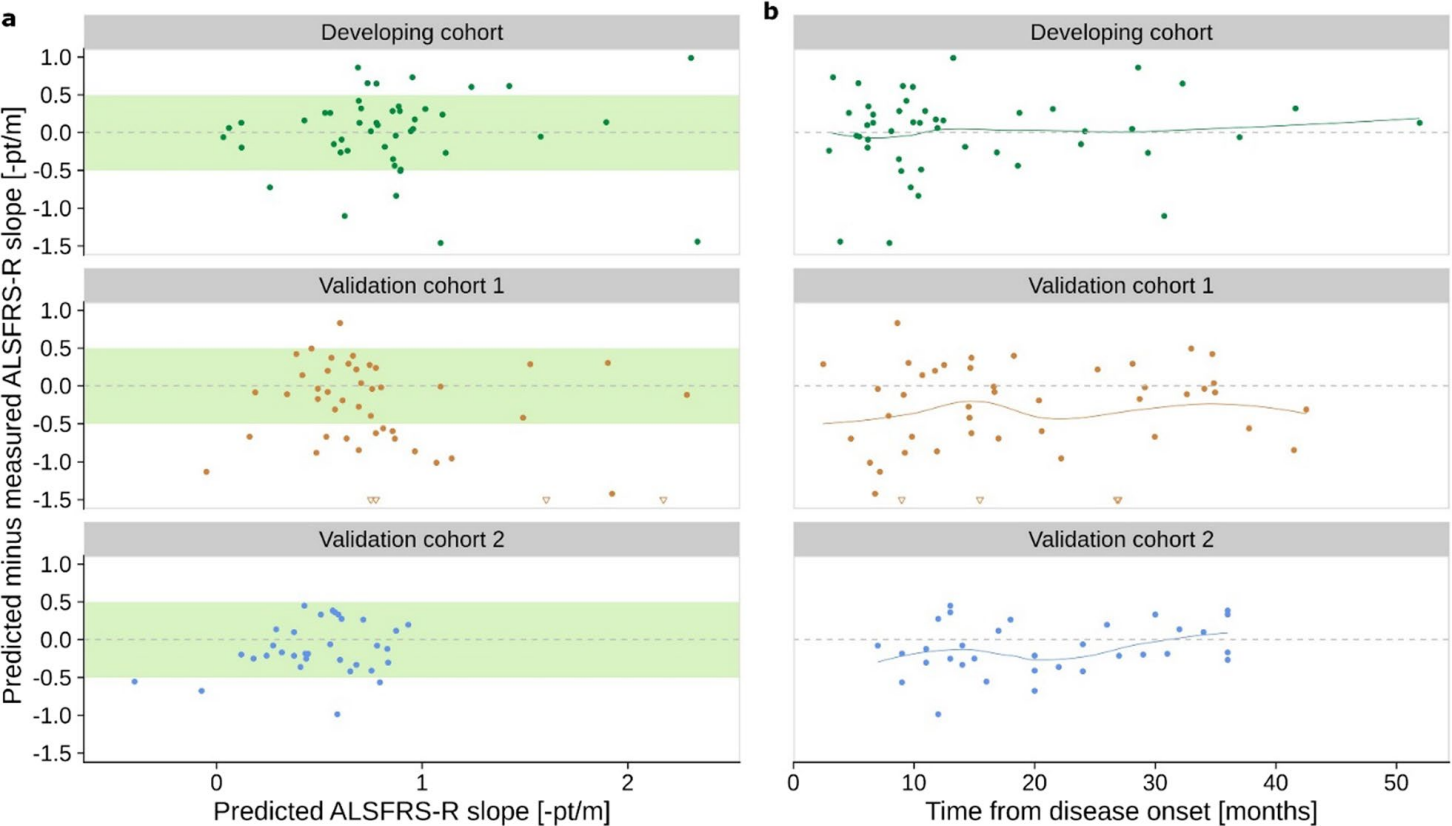
NfL Model Transferability. The absolute deviations of the predicted ALSFRS-R slope using the NfL model from the observed ALSFRS-R slope are plotted against the predicted value (**a**) and the time between disease onset and NfL measurement (**b**). The NfL model predictions use coefficients from the developing cohort, as shown in Fig. 3. Triangular arrowheads indicate points outside the coordinate system, and points inside the green box represent predictions within 0.5 pt/m from the measured value. Colored lines in panel **b** show local regression

As illustrated in Fig. 5a, the model could predict the correct ALSFRS-R slope with less than 0.5 pt/m error for 72%, 59%, and 89% of patients in the DC/V1/V2 cohorts, respectively. Furthermore, absolute deviations were randomly scattered around zero, indicating that the NfL model can be applied equally for patients with low and high progression rates. Importantly, we also did not observe a systematic pattern of deviation concerning disease duration (Fig. 5b).

**Fig. 6:**
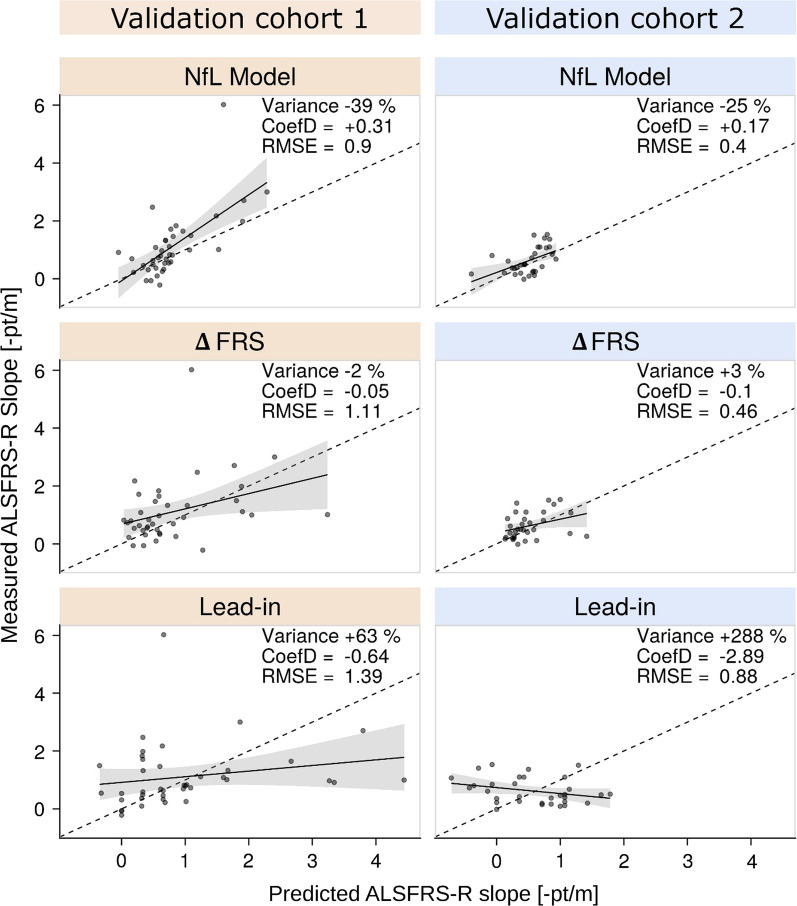
Predictive performance of the NfL model in comparison to the ΔFRS and the lead-in approaches. The scatter plots show predicted versus measured ALSFRS-R slopes for the interventional period of a simulated clinical trial in the validation cohorts V1 (*n* = 40) and V2 (*n* = 33). Trend lines with grey areas (standard error) visualize systematic deviation from the perfect prediction (dashed lines). For each method and cohort, the change of variance, and RMSE and CoefD values are provided in the upper right corner. Note that the RMSE represents absolute values and can only be compared with one another within the same data set; the smaller the RMSE, the more precise the prediction. CoefD can range from − ∞ to 1, with the value of 1 meaning perfect prediction, positive values indicating the model adds predictive information, while negative values indicating the opposite. A decrease in variance indicates an increase in statistical power in a clinical trial

Figure 6 compares the measured ALSFRS-R slopes to the predictions computed with the NfL model, the ΔFRS method, and the lead-in period. The NfL model added valuable information in both validation cohorts, as indicated by the lowest RMSE, positive CoefD values, and a considerable decrease in slope variances. In contrast, we observed negative CoefD, high RMSE, and increased variance for the lead-in approach. Using ΔFRS-based predictions, the CoefD values and variance change remained close to zero.

In line with these findings, the mixed-effects models showed trial size reductions for the NfL model of 61% in V1 (95%CI 54%–66%) and 22% in V2 (95%CI 7%–29%). In contrast, we observed only modest savings for ΔFRS (V1: 12%, 95%CI 1%–17%; V2: 6%, 95%CI − 11% to 12%) and the lead-in period approaches (V1: 6%, 95%CI − 6% to 11%; V2: 2%, 95%CI − 15% to 7%).

### How to apply the model in clinical trials to improve study power

The prediction model could be applied to compensate for heterogeneity of disease progression in a clinical trial with ALSFRS-R as the outcome parameter, using the follows steps:

1. Assessment of site of disease onset (bulbar or spinal) and measurement of NfL in serum or plasma at study baseline (If the measurements are performed with a different assay or if different pre-processing is used, a conversion factor may have to be established);

2. Insert the parameters into the prediction formula we developed:

7 $$\text{ALSFRS-R slope}=4.45-1.13 \ln(\text{NfL})-3.82\,S+0.83\,S\ln(\text{NfL})$$

(*S* = 1 for spinal, *S* = 0 for bulbar), to compute the predicted ALSFRS-R slope for each participant of the study;

3. For each participant, compute the ALSFRS-R slope actually observed throughout the study;

4. For each participant, compute the difference between the ALSFRS-R slope observed throughout the study and the ALSFRS-R slope predicted using the formula;

5. Compare the mean or median difference between observed and predicted ALSFRS-R slope in the placebo and the active treatment groups.

As an alternative to steps 4 and 5, the linear mixed model as described in the methods can be implemented, and coefficient beta2 (interaction of treatment effect and time slope) be tested for significance.

In a conventional study, only the mean or median progression rate of the active treatment and placebo groups can be compared, which are strongly dependent on the natural course of the disease—a therapeutic effect must first overcome the natural heterogeneity of ALS in order to become visible. This reduces the statistical power of a conventional study.

Using the prediction model, it is possible to anticipate the natural course of the disease and include it in the analysis—this significantly increases the study's statistical power.

## Discussion

Interventional trials in ALS suffer from heterogeneity of the disease, which considerably reduces the statistical power of the trials [8–10]. Predictive models that take into account disease heterogeneity are considered a promising tool to meet this challenge but have hardly been established to date [17–21]. In this study, we developed an NfL-based prediction model for monthly decrease of ALSFRS-R score and demonstrated its applicability and impact on trial power.

We showed that adding NfL levels to multivariate predictive models significantly and consistently increased their predictive performance compared to models restricted to clinical parameters. Küffner et al*.* [18] have tested a combination of complex prediction models for ALSFRS-R slopes that incorporated hundreds of clinical variables and basic laboratory parameters—but not neurochemical biomarkers. The predictive quality of this approach is similar to the accuracy provided by blood NfL as a standalone marker [24–28]. This, together with our findings, highlights the predictive value of NfL, especially in comparison to clinical parameters. Interestingly, we found that the NfL-based predictions could be improved by a logarithmic transformation of NfL levels and by including the interaction between NfL levels and the site of disease onset. Our results are consistent with a recent finding that NfL could be used to increase the accuracy of a model predicting progression rates [20]. In addition, we further confirmed the finding of previous studies that blood NfL levels measured longitudinally are steady on a patient level in a timeframe most relevant for interventional trials [20, 24–26, 36]. In summary, these results show that the Nfl-based predictive models meet the basic requirements for application in clinical studies.

Importantly, we demonstrated that the NfL-based prediction model is transferable to new datasets and that the predictions do not systematically depend on the progression rate or disease duration. Using mixed-effects models to simulate randomization and treatment effects of an actual trial, we observed that the prediction model could significantly increase the statistical power by up to 61%. As far as we know, only two previous studies had quantified the impact of prediction models for ALS disease progression on the statistical power. Küffner et al. [18] reported a 20% increase of trial power using the above-mentioned combination of complex models without neurochemical biomarkers. In contrast, a recently published NfL-based prediction model only yielded a modest increase of 8% in the trial power [20]. However, this model was developed incorporating a relatively large proportion of patients in later disease stages and thus tended to underrepresent patients with faster disease progression [20]. By developing our NfL model in patients at the time of diagnosis, we aimed to base the model on the broadest possible progression spectrum and supposed that this had a crucial effect, though, at the same time, it reduced the number of evaluable patients. In summary, our results can serve as a proof of concept for the use of NfL-based prediction models to target the challenge of disease heterogeneity in interventional trials.

Eventually, the application of the NfL-based model is only useful if it is advantageous over the strategies currently used. By analyzing the predictive value of our NfL model in direct comparison to the ΔFRS and a lead-in period approaches used in recent trials, we observed superiority of the NfL-based approach [30–32, 37–39]. Surprisingly, we also found that a lead-in period was not a reliable method to anticipate disease progression in our cohorts. Although there could be other reasons to conduct a lead-in period, predicting the ALSFRS-R course seems not to justify the delay in starting the intervention and the costs incurred [40]. Replacing a lead-in period by a single NfL measurement would result in a shorter interval from disease onset to therapy, potentially being favorable to detect a drug's efficacy because of less advanced motorneuron degeneration [9].

Our study has some limitations. Although the model shows a reliable test performance at the group level, we observed clinically relevant deviations in some patients, making the model less suitable for individual predictions. The predictive accuracy might be further improved by incorporating respiratory parameters and genetic status or by adjusting for confounders of NfL levels such as age and related morphologic brain changes [41], renal function, and blood volume [41–43]; however, the data available for our cohorts did not allow further investigations. Methods to reduce the noise of the ALSFRS-R itself during assessment might also enhance the development of prediction models [1, 44–46]. Here, we restricted our analyses to a linear model as it facilitates direct interpretability of the influence of the candidate predictors on the accuracy of the prediction. Non-linear and non-parametric approaches are harder to interpret but might outperform linear models in terms of accuracy [7, 47] and hence would be an interesting topic for future studies. An additional topic for future research could be predictive models for survival, which could be used for studies with survival as the primary endpoint or for reasons of stratified randomization and anticipation of dropouts. An exploratory analysis of the utility of NfL for this purpose is provided in Additional file 3. A further limitation is the relatively small sample size in each cohort. Multicenter evaluation in future studies or incorporation of the prediction model in actual phase 3 trials could help gain more insights into the strengths and limits of the model. Due to the limited blood sample availability for model validation, the validation cohorts represent only an excerpt of the original placebo cohorts. In the V2 cohort, this caused a shift to younger patients with a rather slow disease progression and revealed that the NfL model may perform better in cohorts including a broader progression spectrum. Eventually, the blood samples in the V2 cohort were EDTA plasma, while the DC and V1 cohorts provided serum samples. Although the NfL assay is equally usable for serum and EDTA-plasma and quality controls did not reveal deviations exceeding the inter-assay coefficients of variability, it represents a potential confounder.

## Conclusions

Blood NfL is a valuable prognostic biomarker. Using the NfL-based prediction model to compensate for clinical heterogeneity could considerably improve the trial power and help distinguish treatment effects from the inter-individual variance of disease progression in future randomized controlled trials. The complementary implementation of NfL-based prediction models in ALS trials could provide further insights into the possible applications.

## Supplementary Information

**Additional file 1** contains the longitudinal data of each patient in the study (ALSFRS-R sum score, NfL level in pg/ml).

**Additional file 2** contains the clinical data of each patient in the study (patient, cohort, onset, gender, age, FRS, NfL levels at baseline, ALSFRS-R at baseline, BMI at baseline, deceased status, months survived since disease onset).

**Additional file 3** Exploratory analysis of blood NfL’s impact on multivariate regression models predicting survival.

## Data Availability

All data generated or analysed during this study are included in this published article and its Additional files.

## Abbreviations

ALS: Amyotrophic Lateral Sclerosis
ALSFRS-R: Amyotrophic Lateral Sclerosis Functional Rating Scale Revised
CoefD: Coefficient of Determination
DC: Development cohort
NfL: Neurofilament light chain
RMSE: Root-mean-square error
V1: Validation cohort 1
V2: Validation cohort 2
ΔFRS: Monthly ALSFRS-R decrease since disease onset
