# Supplementary material for: Neurofilament light and heterogeneity of disease progression in amyotrophic lateral sclerosis: development and validation of a prediction model to improve interventional trials
Source: Transl Neurodegener. 2021 Aug 26;10:31. doi: 10.1186/s40035-021-00257-y (PMC8390195; doi:10.1186/s40035-021-00257-y)
Supplement: Supplementary file 3 — Additional file 3 Exploratory analysis of blood NfL’s impact on multivariate regression models predicting survival. [file 40035_2021_257_MOESM3_ESM.pdf]

Supplement

Neurofilament Light and Heterogeneity of Disease Progression in Amyotrophic Lateral Sclerosis: Development and Validation of a Prediction Model to Improve Interventional Trials

Exploratory analysis of blood Neurofilament Light's (NfL) impact on multivariate regression models predicting survival

Introduction:

Precise predictive models for survival could be a complementary tool for future interventional trials in Amyotrophic Lateral Sclerosis (ALS). They could be used to increase statistical power in studies with survival as the primary endpoint or for reasons of stratified randomization and anticipation of dropouts. Although our study focuses and hence was designed to develop and validate a model predicting disease progression, we aimed to explore the benefit blood NfL could provide for the prediction of survival.

Methods:

For survival analyses, we pooled the data from the three cohorts used in our study. Overall, 32 patients (26%) of all participants were followed up to death. This group consists of 10 patients with spinal onset ALS from the development cohort (DC), 14 patients with spinal onset as well as 8 patients with bulbar onset from validation cohort 1 (V1).

We fitted a multivariate linear regression model with the same candidate predictors used for the disease progression model: baseline ln(NfL), sex, age, site of onset (bulbar or spinal), body mass index (BMI), monthly ALSFRS-R decrease between disease onset and baseline (ΔFRS), and ALSFRS-R score at baseline. We again used sequential F-tests to eliminate non-significant predictors one by one, only this time we used overall survival (in months since diagnosis) instead of the ALSFRS-R slopes.

Additionally, we included all patients of the pooled cohort in a Kaplan Meier analysis for survival since disease onset with subgroups defined as NfL baseline above the median (>86 pg/ml) or below the median (≤86 pg/ml). The log-rank test was used to test for significance. Patients not deceased throughout follow-up were censored at the time of the last visit.

Results and Discussion:

Evaluating the impact NfL could have on multivariate regression models for survival, we found that including ln(NfL) consistently improved model performance ($p<0.05$, F-test) (**Supplement Table 1**). In contrast to the prediction model for ALSFRS-R slopes, overall survival was significantly correlated with ΔFRS. ΔFRS and ln(NfL) were statistically significant in all models we tested. Given the small number of deceased patients (n=32), the analysis presented here is intended as exploration. Still, we find survival is significantly correlated with NfL blood levels at baseline.

Supplement Table 1. Impact of NfL on Survival Models

**Predictors and p-values**<br>(˙ $P < 0.1$, * $P < 0.05$, ** $P < 0.01$)	**Correlation**<br>(square root of adjusted R^2)	**Correlation for same model without ln(NfL)**
**Including all candidate predictors**<br>ln(NfL) 0.01 **<br>site of onset 0.08 ˙<br>ALSFRS-R score 0.02 *<br>age at onset 0.16<br>**Δ**FRS 0.01 **<br>sex 0.52<br>BMI 0.69<br>ln(NfL):site of onset 0.54<br>ln(NfL):sex 0.05 *	**0.66**	**0.59**
**After removing BMI**<br>ln(NfL) 0.01 **<br>site of onset 0.06 ˙<br>sex 0.56<br>ALSFRS-R score 0.05 *<br>age at onset 0.25<br>**Δ**FRS 0.01 **<br>ln(NfL):site of onset 0.47<br>ln(NfL):sex 0.05 *	**0.65**	**0.58**
**After removing age at onset and NfL:site of onset**<br>ln(NfL) 0.01 **<br>site of onset 0.06 ˙<br>sex 0.56<br>ALSFRS-R score 0.05 *<br>**Δ**FRS 0.01 **<br>ln(NfL):sex 0.04 *	**0.66**	**0.58**
**After removing sex**<br>ln(NfL) 0.01 **<br>site of onset 0.07 ˙<br>ALSFRS-R score 0.05 *<br>**Δ**FRS 0.01 **	**0.61**	**0.59**
**After removing site of onset**<br>ln(NfL) 0.01 **<br>ALSFRS-R score 0.07 ˙<br>**Δ**FRS 0.01 **	**0.58**	**0.53**
**After removing ALSFRS-R score**<br>ln(NfL) 0.02 *<br>**Δ**FRS 0.05 *	**0.45**	**0.41**

Table legend: : = interaction between candidate predictors

A Kaplan Meier analysis for survival since disease onset with subgroups defined as NfL baseline above median (>86 pg/ml) or below median (≤86 pg/ml) showed significantly better survival in patients with low baseline NfL levels ($P < 0.001$, **Supplement Figure 1**). The Kaplan Meier results fit the results of previous studies performing similar analyses (1-4), confirming that NfL is a strong predictor for survival in patients with ALS.

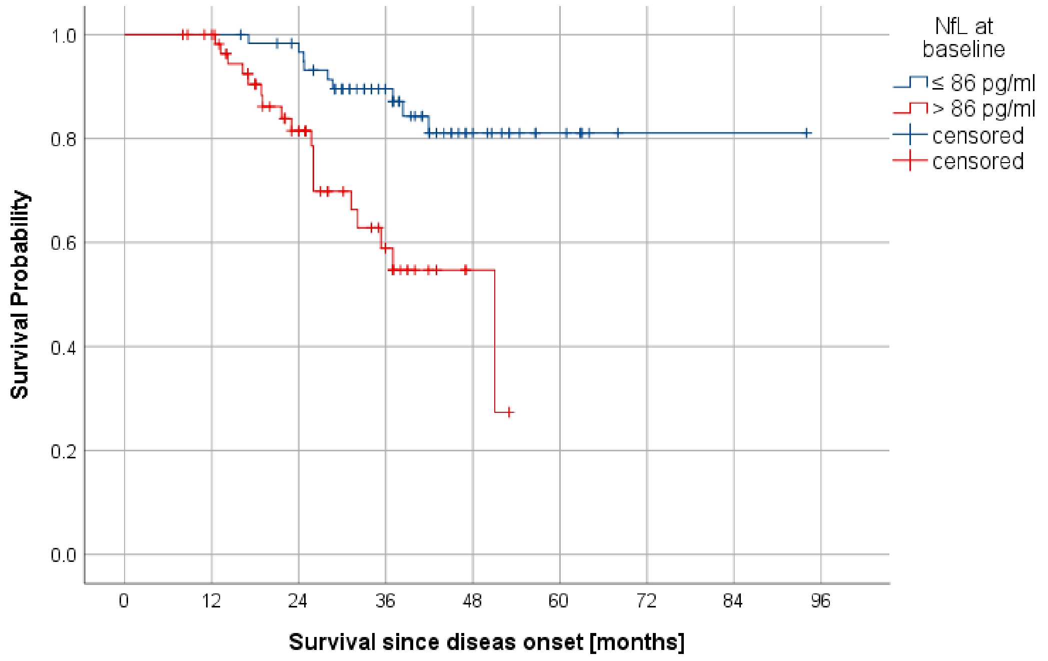

Supplement Figure 1. Kaplan Meier

Kaplan Meier plot for survival since disease onset for patients split according to their baseline NfL levels. If not deceased, patients were censored at last visit. Patients with NfL baseline levels below median (≤ 86 pg/ml; blue) showed significantly longer survival ($P < 0.001$, log-rank) than patients with baseline NfL levels above median (> 86 pg/ml, red).

Conclusion:

Our exploration points toward blood NfL at baseline being a promising biomarker to improve models predicting survival in ALS. Due to the low number of deceased cases in our study, results are intended only as exploration for future research.

Supplement References

(1) Verde F, Steinacker P, Weishaupt JH, Kassubek J, Oeckl P, Halbgebauer S, et al. Neurofilament light chain in serum for the diagnosis of amyotrophic lateral sclerosis. J Neurol Neurosurg Psychiatry 2019;90(2):157-164.

(2) Thouvenot E, Dematteі C, Lehmann S, Maceski-Maleska A, Hirtz C, Juntas-Morales R, et al. Serum neurofilament light chain at time of diagnosis is an independent prognostic factor of survival in amyotrophic lateral sclerosis. European Journal of Neurology 2020;27(2):251-257.

(3) Lu C, Macdonald-Wallis C, Gray E, Pearce N, Petzold A, Norgren N, et al. Neurofilament light chain. Neurology 2015 Jan 1,;84(22):2247-2257.

(4) Steinacker P, Huss A, Mayer B, Grehl T, Grosskreutz J, Borck G, et al. Diagnostic and prognostic significance of neurofilament light chain NF-L, but not progranulin and S100B, in the course of amyotrophic lateral sclerosis: Data from the German MND-net. Amyotroph Lateral Scler Frontotemporal Degener 2017 Feb;18(1-2):112-119.
